# A Little Holiday in the Spring

**Published:** 1887-04-02

**Authors:** 


### A LITTLE HOLIDAY IN THE SPRING.

People in London, and other large towns, ought to study the art of healthy living. Unless a certain standard of health be pretty constantly maintained, there is very little pleasure in life, and not so much goodness as there ought to be. Life ought to be, and for most people can be, fairly pleasant. No doubt many are compelled to live "with their noses to the grindstone," as it is forcibly expressed; but even these should try to arrange a little occasional pleasure for themselves whenever it is possible. The French say we "take our pleasures sadly." That is not very important provided we do take them, and that they are to us real pleasures.

Large towns generally, and London especially, make great demands upon our powers. The town man soon becomes conscious that town life is not a holiday business. There is a feeling of strife and struggle in the very atmosphere. A man speedily recognises that he must use his eyes and his ears, his hands and his elbows, his head and his feet. He must either make courageous and determined efforts to be in the front of the race, or else he will be quickly and inextricably entangled amongst the thousands of incapables who crowd in the rear. But the race is long; and the man who runs and wins *long* races is the *trained* man. The raw lout has no chance against the expert. The skilled and experienced runner of long races does not start off at ten miles an hour, and try to keep up that pace till the end of the course. He starts leisurely, and takes a rest at appointed places. This is emphatically what the really intelligent man will do in the race of life. Some people say they cannot be spared from their business. It will fall into arrears whilst they are away. Probably it may; but if they keep to their business too constantly, the business may go ahead, but they themselves will get into arrears. Their health, their livers, their kidneys, their hearts, their brains, their muscles. So that it becomes a question, whether is the more important, the man or his business, the food or the eater, the dog or his tail?

People do not consider that they were made for physical labour, energetic and constant muscular movement. The desk, the shop, the study, the consulting-room—these all mean absolutely unnatural modes of life, so unnatural that unless they are adequately counterbalanced, they infallibly end in physical and mental weakness, and loss of manhood. One method, and a most important one, of counterbalancing these unnatural methods of living is a holiday. A holiday—the delight of schoolboys and of all men of sense. The prig, and the miser, and the blockhead may sneer at holidays. The wise man loves them and takes as many as he reasonably can. No man works so hard and so well as he who revels in a holiday. No man so intensely enjoys a holiday as he who strains his powers to the utmost when he is at work. Unbend the bow now and again, and when it is bent it will send the arrows home with redoubled force. Keep it always bent, and very soon the shaft will fall idly at your feet. Most Londoners take a summer holiday, but only a few indulge in such a luxury in the spring. They cannot afford the time and the money, they think. If they would spend their money more wisely in the winter, go less to the theatre, give fewer dinners, and other parties, and ask their wives and daughters to wear plainer bonnets and less expensive dresses and boots and gloves, the money difficulty would be easily overcome. And if the money pressure were relieved, the time pressure would be taken away at the same time. For, after all, it is because we think we are compelled to earn and spend a certain amount of money every year, that we stick so steadfastly and continuously to our exacting business. To numbers of men a little holiday in the spring would make all the difference between sound health and gradually encroaching disease. Many a man who has died at sixty-five might have lived comfortably till eighty, if he had but possessed a little more good common sense. It is really a matter of sense in the long run. Can a man take an intelligent and judicial view of his own affairs, and can he resolve to do what will be the wisest and best for twenty years to come? Or is he so stupid and feeble-minded, that he allows his business and his home expenses to become complete masters of him? It all lies in the answers to these two questions.

If a Londoner wants to be well and happy, and he ought to want to be both; and if he wishes to give the world the very best that is in him, he must frequently get away from the shadow of St. Paul's. The bricks and the mortar, and the streets, and the cabs, and the omnibuses, and above all the offices with the people who frequent them, must be got out of the brain, and heart, and senses as thoroughly as possible. Instead of them must come in the green fields and the hedgerows; the flowers and the trees; the lambs and the birds; the sea and the mountains; the clear sky and the fresh breezes; the sun, and the moon, and the stars. Then comes boyhood back again. Then merely to live is pleasure. Then the dull brain, the panting heart, the wheezing lungs, and the sluggish liver, all eagerly seize their share of the purified blood and the fresh nerve-force, and set up an instant rivalry which shall best fulfil its allotted duty.

No more pills or potions wanted; no more liver pads or waistbands, no more stethoscopes at your chest, or thermometers in your axilla. A pleasant defiance is given to the doctor, and a happy welcome to the cook. If a Londoner wants to get rid of the enfeebling and miserable effects of the winter's fogs and the winter's business, and to prepare himself for the stifling heats and poisonous atmosphere that await him in the summer, let him, by all means, arrange for himself and his family "A Little Holiday in the Spring."

